# CLINICAL TOOLS TO ADDRESS BRAIN HEALTH CONCERNS IN THE CONTEXT OF LATER-LIFE DEPRESSION

**DOI:** 10.1093/geroni/igac059.1090

**Published:** 2022-12-20

**Authors:** Kelly Bergstrom

**Affiliations:** University of Missouri-St. Louis, St. Louis, Missouri, United States

## Abstract

GSA’s revised KAER Toolkit for Primary Care Teams (Fall, 2021) is an important resource, yet the complexities of depressive and cognitive symptoms in aging individuals create particular challenges for generalist behavioral health providers. Mental health practitioners and their patients can benefit from evidence-based clinical intervention materials that address the intersection of depression and brain health concerns. This presentation highlights treatment strategies and clinical tools from the new Brain Health module of the revised 2021 client workbook Treating Later-Life Depression: A Cognitive Behavioral Therapy Approach from Oxford University Press. Examples will be presented of large print within-session “Learn pages” that inform both providers and patients about normative cognitive aging and ways in which cognitive functioning can be affected by depression. Between-session “Practice forms” will also be demonstrated that address lifestyle factors to promote brain health, consideration of whether to complete a cognitive evaluation, and strategies to manage brain health changes.

